# Serum Anti-Müllerian Hormone Is Significantly Altered by Downregulation With Daily Gonadotropin-Releasing Hormone Agonist: A Prospective Cohort Study

**DOI:** 10.3389/fendo.2019.00115

**Published:** 2019-02-26

**Authors:** Panagiotis Drakopoulos, Arne van de Vijver, Jose Parra, Ellen Anckaert, Johan Schiettecatte, Christophe Blockeel, Martin Hund, Wilma D. J. Verhagen-Kamerbeek, Ying He, Herman Tournaye, Nikolaos P. Polyzos

**Affiliations:** ^1^Centre for Reproductive Medicine, Universitair Ziekenhuis Brussel, Vrije Universiteit Brussel, Brussels, Belgium; ^2^Faculty of Medicine and Pharmacy, Vrije Universiteit Brussel, Brussels, Belgium; ^3^CleanicData, Leuven, Belgium; ^4^Roche Diagnostics International Ltd., Risch-Rotkreuz, Switzerland; ^5^Roche Diagnostics GmbH, Penzberg, Germany; ^6^Department of Reproductive Medicine, Dexeus University Hospital, Barcelona, Spain; ^7^Department of Clinical Medicine, Faculty of Health, Aarhus University Incuba, Aarhus, Denmark

**Keywords:** Elecsys® AMH, *in vitro* fertilization, ovarian reserve, ovarian response, GnRH agonist

## Abstract

**Research Question:** What is the effect of gonadotropin-releasing hormone (GnRH)-agonist treatment on serum anti-Müllerian hormone (AMH)?

**Design:** This prospective cohort study conducted in a tertiary university hospital comprised patients (*n* = 52) who self-administered daily triptorelin (0.1 mg/0.1 mL) subcutaneously for 14 days from menstrual cycle day 21 ± 3, between July 2015 and March 2016. Enrolled women were 18–43 years old, considered normal ovarian responders, with a planned GnRH agonist controlled ovarian stimulation protocol. The primary endpoint was to evaluate the effect of GnRH agonist on serum AMH levels after 7 and 14 days of treatment.

**Results:** Under GnRH agonist treatment, serum AMH was significantly decreased vs. baseline on day 7 (mean change from baseline: −0.265 ng/mL; 95% confidence interval [CI], −0.395 to −0.135 ng/mL; *p* < 0.001). On day 14, serum AMH was significantly increased (mean change from baseline: 0.289 ng/mL; 95% CI, 0.140–0.439 ng/mL; *p* < 0.001). Although the median change in AMH from baseline was only −14.9% on day 7 and +17.4% on day 14, from day 7 to 14 AMH significantly increased by 0.55 ng/mL (43.8%; *p* < 0.001), which is of paramount clinical importance. A linear, mixed-effect model demonstrated that GnRH agonist treatment for 7 and 14 days had a highly significant effect on serum AMH concentration after adjustment for confounding factors (age, body mass index, baseline antral follicle count, and visit). AMH assay precision was excellent (four aliquots/sample); coefficient of variation was 1.2–1.4%.

**Conclusions:** GnRH agonist treatment had a clinically significant effect on serum AMH, dependent on treatment duration. The clear V-shaped response of AMH level to daily GnRH agonist treatment has important clinical implications for assessing ovarian reserve and predicting ovarian response, thus AMH measurements under GnRH agonist downregulation should be interpreted with great caution.

## Introduction

Reproductive medicine has advanced and outcomes have improved based on technological progress in equipment and laboratory testing in the last decade. Ovarian reserve and response to controlled ovarian stimulation can now be better assessed, resulting in improved informed clinical decision making and counseling of patients seeking advice on reproductive treatment (1, 2). Numerous biomarkers have been evaluated to assess ovarian reserve and predict ovarian response, however, none of these are able to provide a direct marker of ovarian reserve. In this context, serum anti-Müllerian hormone (AMH) has become popular among clinicians and is widely used in reproductive medicine (3–7). AMH is produced by the granulosa cells of pre-antral and small antral follicles, suggesting that AMH has an important role in folliculogenesis (8).

However, caution is needed when interpreting results, as certain medications and hormones can affect serum AMH levels. Previous studies have demonstrated that current or past use of oral contraceptives is associated with reduced serum AMH (9, 10), with the effect being described as transient and potentially due to the altered development of antral follicles by downregulation of the hypothalamic-pituitary ovarian axis (10, 11). Similarly, the use of gonadotropin-releasing hormone (GnRH) agonist downregulation in women of reproductive age has demonstrated that AMH levels are significantly affected (12, 13). Nonetheless, it should be stated that although the aforementioned treatments may alter AMH levels, they may not be directly detrimental to the ovarian reserve. Conversely, they may only perturb the physiology of the ovary, causing a transient derangement of the complex and unknown mechanisms regulating AMH production.

Over the last 10 years, various AMH assays have been developed, among which the recently introduced fully automated assays appear to demonstrate greater reliability of AMH measurement compared with manual assays. This is supported by accumulating evidence, which demonstrates that automated assays are more strongly correlated with antral follicle count (AFC) in the subset of patients with reduced follicle count (14), and appear to have an improved performance for ovarian response prediction compared with manual assays (15). Despite the excellent analytical performance of new fully automated assays (16), derived AMH values are substantially lower than those obtained by manual assays, with assay-specific interpretation required for routine clinical use (15).

Considering the widespread use of automated AMH assays, and the profound differences in derived values compared with manual assays, it is unclear whether medications such as oral contraceptives or GnRH agonists may have a clinically significant effect on serum AMH levels, when measured with the new assays. The objective of this prospective cohort study was to evaluate the effect on serum AMH of daily administration of a GnRH agonist, for 14 days preceding controlled ovarian stimulation, measured using the fully automated Elecsys® AMH immunoassay.

## Materials and Methods

### Study Design and Patients

The study was a prospective cohort study conducted in a center for reproductive medicine of a tertiary university hospital (Universitair Ziekenhuis Brussel, Vrije Universiteit Brussel, Brussels, Belgium) between July 2015 and March 2016.

Eligible women were aged 18–43 years and considered normal ovarian responders based on AFC >7 and/or baseline serum AMH ≥1.1 ng/mL. Their treatment plan included ovarian stimulation for *in vitro* fertilization (IVF)/intra-cytoplasmic sperm injection using a long GnRH agonist protocol. Women with an AFC ≤ 7 and an AMH level < 1.1 ng/mL were excluded. Other exclusion criteria were polycystic ovary syndrome [according to the Rotterdam criteria (17)], ovariectomy or previous surgery for endometriosis, or a history of gonadotoxic therapy. Women with contraindications for the use of a GnRH agonist or gonadotropins, a recent history of any current untreated endocrine abnormality, or severe disease requiring regular treatment were also excluded from the study.

### Study Treatment and Visits

Starting on study day 0 (day 21 ± 3 days of the menstrual cycle), all patients self-administered the GnRH agonist triptorelin (0.1 mg/0.1 mL) by subcutaneous injection daily for 14 days.

**Figure 1:**
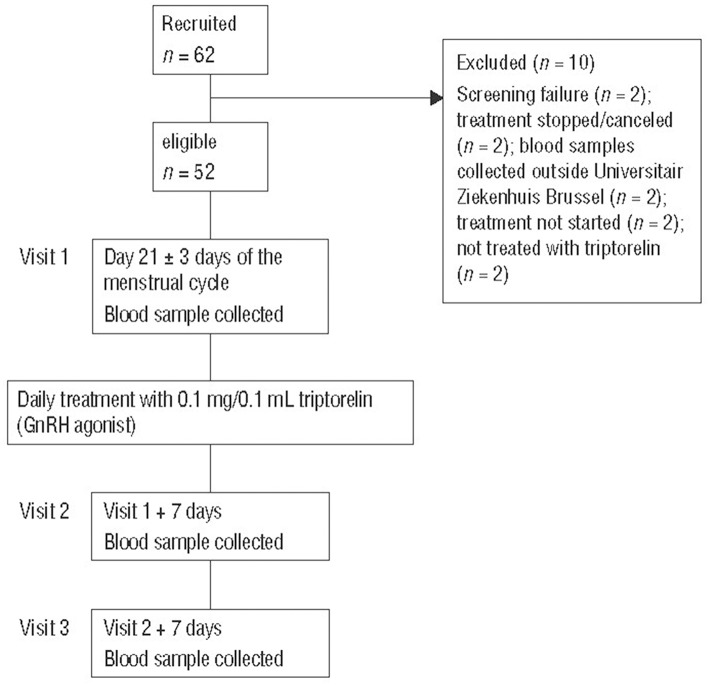
Study design and patient disposition. GnRH, gonadotropin-releasing hormone; UZB, Universitair Ziekenhuis Brussel.

Patients visited the clinic on days 0, 7, and 14 of GnRH treatment (Figure 1). Blood samples for hormone analyses were collected at each visit during the daytime, i.e., before (baseline sample at visit 1 on day 0) and during treatment (visits 2 and 3) with the GnRH agonist.

The primary endpoint of the study was to evaluate the effect of the GnRH agonist on the serum AMH level after 7 and 14 days of treatment. The secondary endpoints were the effect of GnRH agonist treatment on serum levels of follicle-stimulating hormone (FSH), luteinizing hormone (LH), estradiol, and progesterone.

### Hormone Analyses

Venous blood was collected into plain serum tubes and all samples were centrifuged (2–8°C, 2,000 g, 10 min) within 1 h of blood collection to separate the serum. Each serum sample was split into four aliquots. All aliquots were immediately frozen at −80°C and stored frozen until analysis.

In each aliquot, all hormones were analyzed at the same time, and all aliquots from each patient were assessed in a random order in the same run. Each hormone was measured with an Elecsys® assay in conjunction with a **cobas e** 601 module of a **cobas**® 6000 analyzer (Roche Diagnostics, Mannheim, Germany) according to the manufacturer's instructions. The limit of detection of the assays were: AMH, 0.010 ng/mL (0.071 pmol/L); FSH, < 0.100 mIU/mL; LH, 0.100 mIU/mL; estradiol, ≤ 5 pg/mL (≤ 18.4 pmol/L); progesterone, 0.030 ng/mL (0.095 mnol/L). The intermediate precision (coefficient of variation) values were: AMH, 2.7–3.5%; FSH, 3.6–4.5%; LH, 1.6–2.2%; estradiol, 1.9–10.6%; progesterone, 1.8–4.8%.

### Sample Size

The total sample size was 52 subjects, which was needed to achieve 80% power to detect a mean difference of 10% between the AMH baseline value and after 7 or 14 days, with an estimated standard deviation (SD) of the percentage differences of 25 (standardized effect size 0.4), and a significance level (alpha) of 0.05, using a paired *t*-test and assuming that the difference was a normally distributed variable.

### Statistical Analyses

Statistical analyses were performed using software R version 3.0.1. Mean values of each hormone in the four aliquot evaluations were calculated for baseline, day 7, and day 14. Summary statistics (mean, median, SD, percentiles, and range) were calculated and box plots drawn for each hormone for the three study visits. All pairwise absolute (absolute change = visit X–visit 1; X = 2 or 3) and percentage differences (percentage change = [visit X–visit 1]/[visit 1] × 100%) within a patient were calculated for baseline vs. day 7 and day 14 for each hormone.

The mean difference of the hormone levels between baseline and day 7, baseline and day 14, and day 14 and day 7 were compared using a paired *t*-test. The Wilcoxon signed rank test was applied to determine whether the median of the absolute and/or percentage changes from baseline to day 7, from baseline to day 14, and from day 7 to day 14 differed from zero when the changes were not normally distributed.

The primary endpoints were to test whether the mean AMH difference from baseline to day 7 or to day 14 was zero, with a significance level of *p* < 0.05. The secondary endpoints were to explore the changes of FSH, LH, estradiol, and progesterone levels from baseline to days 7 and 14. Significance for the secondary endpoints was adjusted using Bonferroni correction (significance level at 0.0125 = 0.05/4; adjustment for four hypotheses).

A linear, mixed-effect model was performed on AMH repeated measurements with a random slope and random intercept for each patient and day as a fixed effect. This model was used as it is able to handle longitudinal data from the intra-subject measurements of the same clinical biomarker taken at multiple time points. It is an extended linear model, which incorporates fixed and random effects to account for intra-subject variability. The fixed-effect coefficient of day indicates the effect of treatment with GnRH agonist on AMH levels at day 7 and 14 relative to baseline. The random intercept represents the individual variation of AMH baseline, and the random slope indicates the individual slope from the effect of treatment days on AMH levels. The model was adjusted for potential confounding variables of age, body mass index (BMI), and baseline AFC.

## Results

### Patient Disposition and Baseline Demographics

Of the 62 recruited patients, 10 were excluded for the following reasons: screening failure (*n* = 2); treatment stopped/canceled (*n* = 2); blood samples collected outside Universitair Ziekenhuis Brussel (*n* = 2); treatment not started (*n* = 2); not treated with triptorelin (*n* = 2) (Figure 1). Fifty-two patients completed the study treatment and had complete data sets for all study visits.

Patient baseline characteristics are shown in Table 1. The median (IQR) age was 36.0 (33.0–38.0) years. At baseline, the median (IQR) serum AMH concentration was 1.58 (1.03–2.13) ng/mL, and the median (IQR) AFC was 11.0 (9.0–14.0).

**Table 1:** Patient demographics and clinical characteristics.

**Characteristic**	**All patients (*n* = 52)**
Median age (IQR), years	36.0 (33.0–38.0)
Median BMI (IQR), kg/m^2^	23.4 (21.25–25.05)
**RACE**, ***n*** **(% OF TOTAL STUDY POPULATION)**	
White	48 (92.3)
Asian	1 (1.9)
Black	2 (3.9)
Other	1 (1.9)
**SMOKING STATUS**, ***n*** **(% OF TOTAL STUDY POPULATION)**	
Yes	4 (7.7)
No	47 (90.4)
Missing	1 (1.9)
Median AMH (IQR) at baseline, ng/mL	1.58 (1.03–2.13)
Median AFC (IQR) at baseline	11.0 (9.0–14.0)
**AFC CLASS**, ***n*** **(% OF TOTAL STUDY POPULATION)**	
0–7	6 (11.5)
8–15	35 (67.3)
>15	6 (11.5)
Missing	5 (9.6)

*AFC, antral follicle count; AMH, anti-Müllerian hormone; BMI, body mass index; IQR, interquartile range*.

### Effect of GnRH Agonist Treatment on Serum AMH (Primary Endpoint)

**Figure 2:**
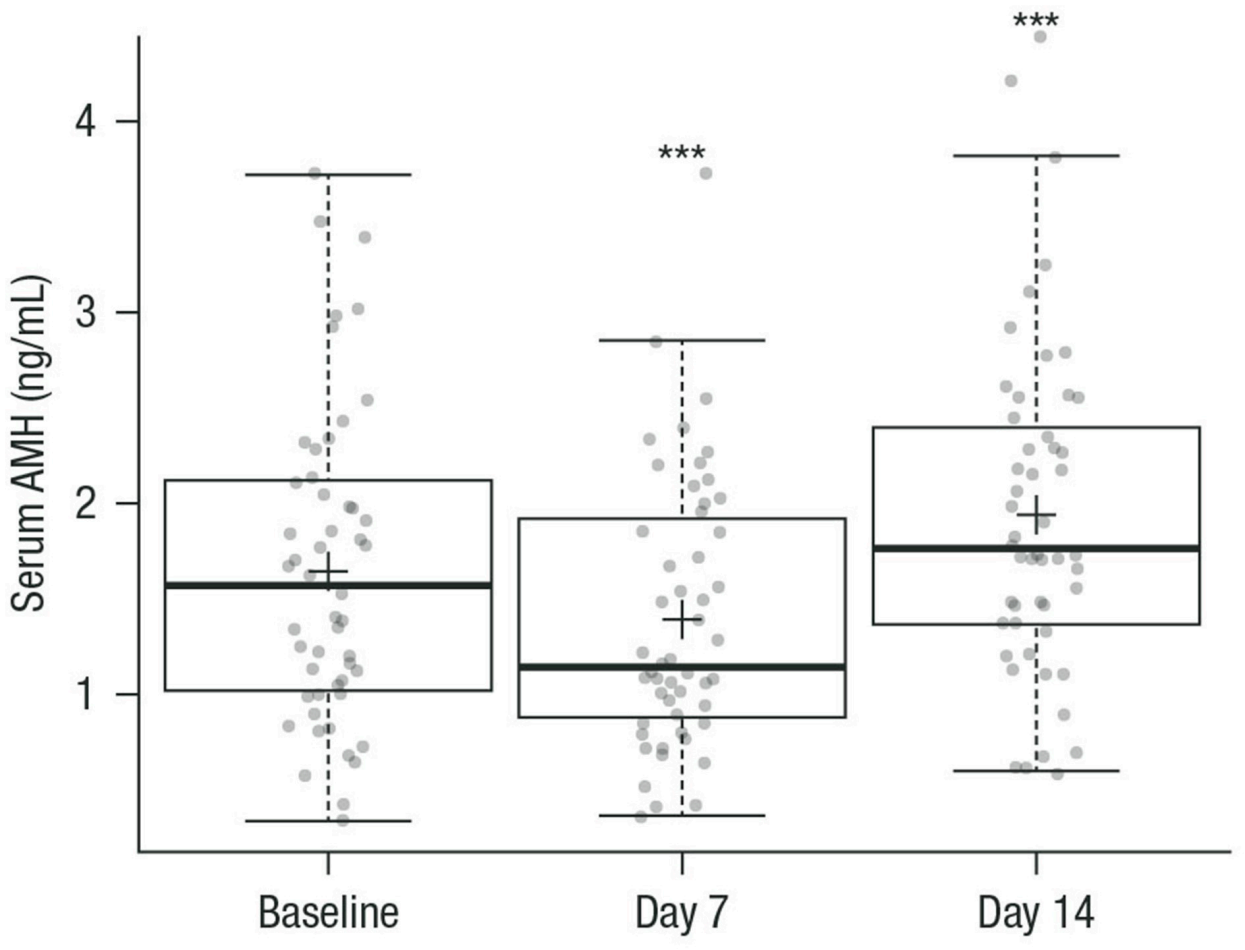
Box plots of serum AMH at baseline (prior to GnRH agonist treatment) and on days 7 and 14 during GnRH agonist treatment. Circles represent individual patient data (mean values calculated from the replicate measurements of the four aliquots for each sample at each visit); crosses are the mean value of AMH of all patients at each visit; horizontal lines summarize the median and the first and third quartiles (within the box) and 1.5x the interquartile range (whiskers). *n* = 52 for all measurements. ^***^p < 0.001 vs. baseline. AMH, anti-Müllerian hormone; GnRH, gonadotropin-releasing hormone.

During GnRH agonist treatment, the serum AMH level followed a V-shape from baseline to day 7 and day 14 (Figure 2).

Supplementary Table S1 summarizes serum AMH levels across the three visits. Box plots for serum FSH, LH, estradiol, and progesterone levels during GnRH treatment are shown in Supplementary Figure S2.

On day 7, the concentration of AMH was significantly decreased; the mean of the absolute change was −0.265 ng/mL (95% confidence interval [CI], −0.395 to −0.135 ng/mL), or −1.89 pmol/L (95% CI, −2.82 to −0.967 pmol/L), with *p* < 0.001 compared with baseline. On day 14, the AMH level was significantly increased with a mean absolute increase of 0.289 ng/mL (95% CI, 0.140–0.439 ng/mL) or 2.07 pmol/L (95% CI, 0.997–3.14 pmol/L) with *p* < 0.001 compared with baseline (Table 2A). On day 14, the mean AMH concentration was significantly increased by 0.55 ng/mL or 3.96 pmol/L with *p* < 0.001 compared with day 7 (Table 2A). The median of the percentage change in AMH values was significantly decreased by 14.9% on day 7 compared with baseline, and significantly increased by 17.4% on day 14 compared with baseline. On day 14, AMH was significantly increased by 48.3% compared with day 7 (Table 2B).

**Table 2:** Statistical analyses of **(A)** absolute and **(B)** percentage change in serum levels of AMH, FSH, LH, estradiol, and progesterone from baseline (prior to GnRH agonist treatment) to days 7 and 14, and between days 14 and 7 during GnRH agonist treatment.

**Hormone^**a**^**	**Day 7 vs. baseline**		**Day 14 vs. baseline**		**Day 14 vs. day 7**	
**A**	**Absolute change 95% CI**	***p*-value^**b**^**	**Absolute change 95% CI**	***p*-value^**b**^**	**Absolute change 95% CI**	***p*-value^**b**^**
AMH, ng/mL	−0.265 −0.395 to −0.135	< 0.001	0.289 0.140–0.439	< 0.001	0.555 0.419–0.690	< 0.001
AMH, pmol/L	−1.89 −2.82 to −0.967	< 0.001	2.07 0.997–3.14	< 0.001	3.96 2.99–4.93	< 0.001
FSH, IU/L	−0.494 −1.08 to 0.094	NS	−0.17 −0.830 to 0.490	NS	0.324 −0.096 to 0.745	NS
LH, IU/L	−0.555 −1.41 to 0.299	NS	−3.02 −3.95 to −2.01	< 0.001	−2.47 −3.20 to −1.73	< 0.001
Estradiol, pg/mL	−16.4 −49.0 to 16.1	NS	−107^c^ −138 to −89.0	< 0.001^c^	−58.6^c^ −124 to −36.9	< 0.001^c^
Progesterone, nmol/L	−3.96 −7.32 to −0.606	NS	−11.1^c^ −13.8 to −8.60	< 0.001^c^	−2.91^c^ −10.9 to −1.03	< 0.001^c^
**B**	**Percentage change 95% CI**	***p*** **value**^**c**^	**Percentage change 95% CI**	***p*** **value**^**c**^	**Percentage change 95% CI**	***p*** **value**^**c**^
AMH	−14.9 −23.0 to −10.1	< 0.05	17.4 10.4–35.9	< 0.001	48.3 34.7–57.4	< 0.001
FSH	−19.8 −27.5 to 3.04	NS	−7.31 −25.0 to 22.6	NS	5.47 −6.97 to 22.6	NS
LH	−9.96 −17.6 to 17.2	NS	−43.2 −55.4 to −33.1	< 0.001	−47.6 −52.2 to −41.0	< 0.001
Estradiol	−28.6 −60.6 to 25.3	NS	−94.8 −95.9 to −93.4	< 0.001	−90.5 −94.5 to −81.0	< 0.001
Progesterone	−69.2 −85.5 to 0.168	NS	−95.8 −97.7 to −94.5	< 0.001	−90.9 −94.7 to −79.1	< 0.001

*AMH, anti-Müllerian hormone; CI, confidence interval; FSH, follicle stimulating hormone; GnRH, gonadotropin-releasing hormone; LH, luteinizing hormone; NS, not significant*.

a *n = 52 for all measurements*.

b *Paired t-test, mean of the absolute change*.

c *Wilcoxon signed rank test, median of the absolute/percentage change. Significant level for AMH was p < 0.05 (primary endpoint). Significant level for the other hormones was p < 0.0125 after Bonferroni correction (exploratory endpoints)*.

Importantly, excellent analytical precision was achieved, demonstrating low variability in the replicate measurements of serum AMH in the four aliquots of the same sample (Supplementary Figure S1). The coefficients of variation for repeatability of the AMH assay were 1.3% at baseline, 1.2% at day 7, and 1.4% at day 14.

### Linear, Mixed-Effect Model for AMH, and GnRH Agonist Treatment

The above statistical findings were confirmed by a linear mixed-effects model (Supplementary Table S2). This model showed that treatment with a GnRH agonist for 7 and 14 days had a highly significant effect on the serum AMH concentration after adjusting for confounding factors of age, BMI, baseline AFC, and visit.

### Effect of GnRH Agonist Treatment on Other Hormones (Exploratory Endpoints)

Supplementary Table S1 lists the serum levels of FSH, LH, estradiol, and progesterone over time. Compared with baseline values, FSH was, on average, 0.494 IU/L lower on day 7 and 0.17 IU/L lower on day 14; however, these changes were not statistically significant (Table 2). Compared with baseline, GnRH agonist treatment had no significant effect on the serum LH, estradiol, and progesterone levels on day 7 (decreased by 0.555 IU/mL, 16.4 pg/mL, and 3.96 nmol/L, respectively), whereas these levels on day 14 were significantly decreased by 3.02 IU/mL, 107 pg/mL, and 11.1 nmol/L, respectively (Table 2).

## Discussion

The present study demonstrates that during GnRH agonist treatment with daily triptorelin injections of 0.1 mg/0.1 mL, serum AMH was statistically significantly decreased on day 7 (by 14.9%) and significantly increased on day 14 (by 17.4%) vs. baseline, with a clear V-shaped response of the AMH level. This V-shaped response of AMH to GnRH agonist treatment was also observed after adjusting for relevant confounders, including age and BMI. Furthermore, AMH was significantly increased by 48.3% on day 14 compared with day 7. Our short-term follow-up data emphasize that AMH levels follow a predictable, biphasic trajectory after GnRH is administered, thereby limiting the utility of AMH as a predictive marker of ovarian response during downregulation.

The findings of the current study follow the same direction as the results of two previous studies, which reported changes in serum AMH with GnRH agonist treatment in women of reproductive age, using older manual AMH assays (12, 13). Nonetheless, they differ substantially on the actual effect size observed. At 7 days after initiation of the downregulation with the GnRH agonist, we observed a smaller median decrease of 14.9% in serum AMH levels compared with the 24% decrease observed by Su et al. (13). At 14 days post-initiation of the agonist, median AMH levels increased by only 17.4% in our dataset, compared with increases of 13 and 32% observed by Su et al. (13) and Jayaprakasan et al. (12), respectively. A potential reason for different effect sizes may be the use of the automated Elecsys® AMH assay, which is associated with higher precision compared with the manual assays Gen II (13) and Diagnostic Systems Lab (12).

Our findings are considered to be robust, as AMH levels were measured using the fully automated Elecsys® AMH immunoassay. In contrast to previous studies (12, 13), multiple aliquots were prepared from each blood draw and measured in the same run, accounting for potential variance and increasing methodological robustness. The rationale behind using an automated assay compared with previously used manual assays was the number of advantages, including a broad linear range, and excellent sensitivity and precision (16). Therefore, it could be stated that the current findings are highly likely to result from the clinical effect of the GnRH agonist on serum AMH and not from variation in the analytical performance of the AMH assay, which may have been the case in the studies mentioned above.

A potential explanation for the significant changes observed in the serum AMH levels may be a direct gonadotropin-independent effect of the GnRH agonist on granulosa cells. There is evidence that GnRH receptors are expressed in human granulosa cells and are up-regulated by GnRH (18). In this regard, the decline of AMH after 7 days of agonist treatment may have resulted from up-regulation of GnRH receptors due to the antiproliferative effects of short-term GnRH agonist on granulosa cells (18). However, it remains unclear why AMH increased after 14 days. In this regard, it should be highlighted that the underlying mechanism of GnRH agonist treatment on serum AMH is currently unknown and requires further investigation.

The present study has several strengths. Firstly, contrary to previous studies, only patients undergoing fertility treatment were included, who are the target population for AMH evaluation, and we adopted a prospective longitudinal design with repeated hormone measurements to ensure that the patients served as their own controls. In this regard, the sample size of our study was large enough to achieve statistical power of 80% to detect an effect of GnRH agonist treatment on serum AMH levels. Secondly, blood-sample collection and processing were standardized in a very robust manner, to reduce potential bias induced by variation in sample pre-analytics, as has already been reported with certain manual AMH enzyme-linked immunosorbent assays (19). Finally, we utilized the fully automated Elecsys® AMH immunoassay, which has been proven to demonstrate excellent sensitivity and precision (16). However, as we only included patients considered as normal responders who were planned to undergo ovarian stimulation by an agonist protocol, our results cannot be extrapolated to other ovarian response categories.

In conclusion, daily GnRH agonist treatment has a statistically and clinically significant effect on the serum AMH levels of infertile women planned to undergo ovarian stimulation. The median change in AMH from baseline was only −14.9% on day 7 and +17.4% on day 14, and a remarkable 48.3% increase in AMH values was observed between day 7 and day 14 of GnRH agonist treatment, which is of paramount clinical importance. Our findings have important clinical implications when predicting ovarian response using AMH as a biomarker. AMH values should be interpreted with great caution, given that AMH values can be significantly altered during GnRH agonist treatment.

## Data Availability

All datasets generated for this study are included in the manuscript and the supplementary files.

## Ethics

This study was earned out in accordance with the recommendations of the UZ Brussel Ethics Committee with written informed consent obtained from all subjects in accordance with the Declaration of Helsinki. The protocol was approved by the UZ Brussel Ethics Committee (BUN: 143201524950).

## Author Contributions

MH, YH, and NP contributed to conception and design (protocol development and/or design advice), data analysis, and data interpretation. EA contributed to data acquisition (hormone analyses) and data analysis. JP and WV-K contributed to data analysis and data interpretation. AvdV (recruiting patients) and JS (sample processing, hormone measurements, and data entry) contributed to data acquisition. NP and PD contributed to the writing of the manuscript and data interpretation. CB, AvdV, and HT contributed to data interpretation. All authors also contributed to the critical review of the important intellectual content of the article and final approval of the version submitted. All relevant datasets generated for this study are included in the manuscript and the supplementary files.

### Conflict of Interest Statement

CB received honoraria and/or research grants from MSD, Ferring, Merck, Abbott, and Besins. MH, WV-K, and YH are employees of Roche. HT has received research grants from Merck-Serono, MSD, Goodlife, Cook, Roche, Besins, Ferring, Research Fund of Flanders (FWO), and Mirtha (now Allergan), and consultancy fees from Finox, Abbott, ObsEva, and Merck. NP received honoraria for lecturing and/or research grants from MSD, Ferring International, Merck, Roche Diagnostics, IBSA, and BESINS International. PD received honoraria for lecturing and/or research grants from MSD, Ferring International and Merck. The remaining authors declare that the research was conducted in the absence of any commercial or financial relationships that could be construed as a potential conflict of interest.

## Abbreviations

AFC: antral follicle count
AMH: anti-Müllerian hormone
BMI: body mass index
CI: confidence interval
FSH: follicle-stimulating hormone
GnRH: gonadotropin-releasing hormone
IVF: *in vitro* fertilization
LH: luteinizing hormone
SD: standard deviation.
