# Supplementary material for: Serum Anti-Müllerian Hormone Is Significantly Altered by Downregulation With Daily Gonadotropin-Releasing Hormone Agonist: A Prospective Cohort Study
Source: Front Endocrinol (Lausanne). 2019 Feb 26;10:115. doi: 10.3389/fendo.2019.00115 (PMC6399150; doi:10.3389/fendo.2019.00115)
Supplement: Supplementary file 1 [file Data_Sheet_1.docx]

Supplementary data

**Supplementary Figure S1**

Individual values for each of the four replicates of serum AMH for each individual patient and at each of the three time points (*n* = 52); AMH, anti-Müllerian hormone; BL, baseline.


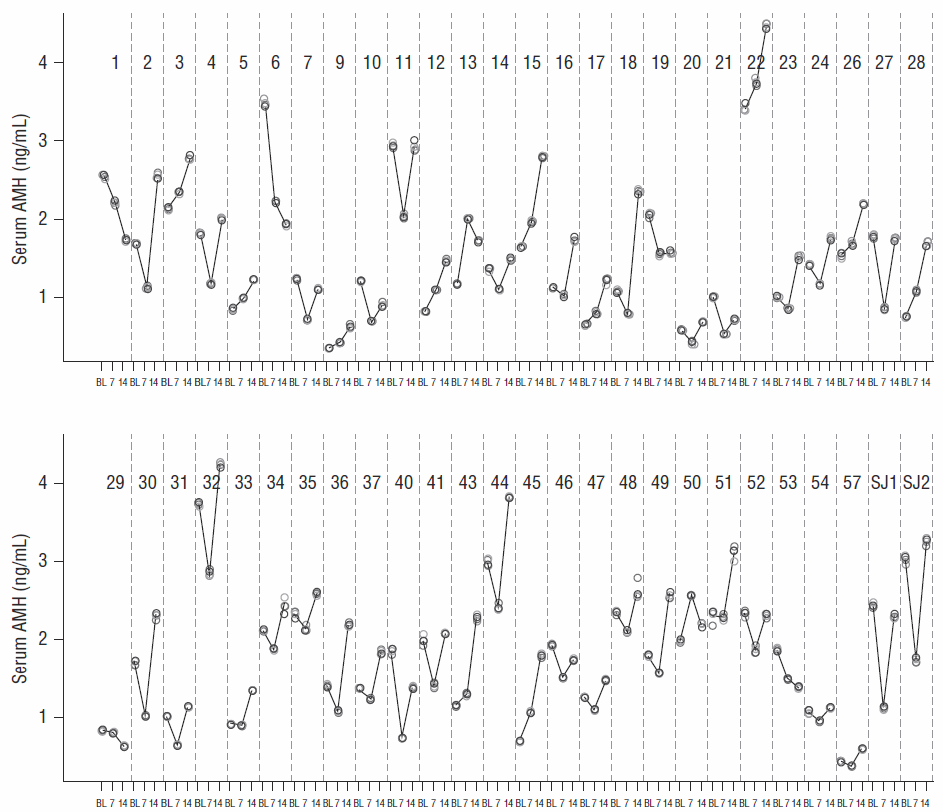


**Supplementary Figure S2**

Box plots of serum (a) FSH, (b) LH, (c) estradiol, and (d) progesterone, at baseline (prior to GnRH-agonist treatment) and on days 7 and 14 during GnRH-agonist treatment. Circles represent individual patient data (mean values calculated from the replicate measurements of the four aliquots for each sample at each visit); crosses are the mean value of all patients at each visit; horizontal lines summarize the median and the first and third quartiles (within the box) and 1.5x the interquartile range (whiskers). FSH, follicle-stimulating hormone, GnRH, gonadotropin-releasing hormone, LH, luteinizing hormone.


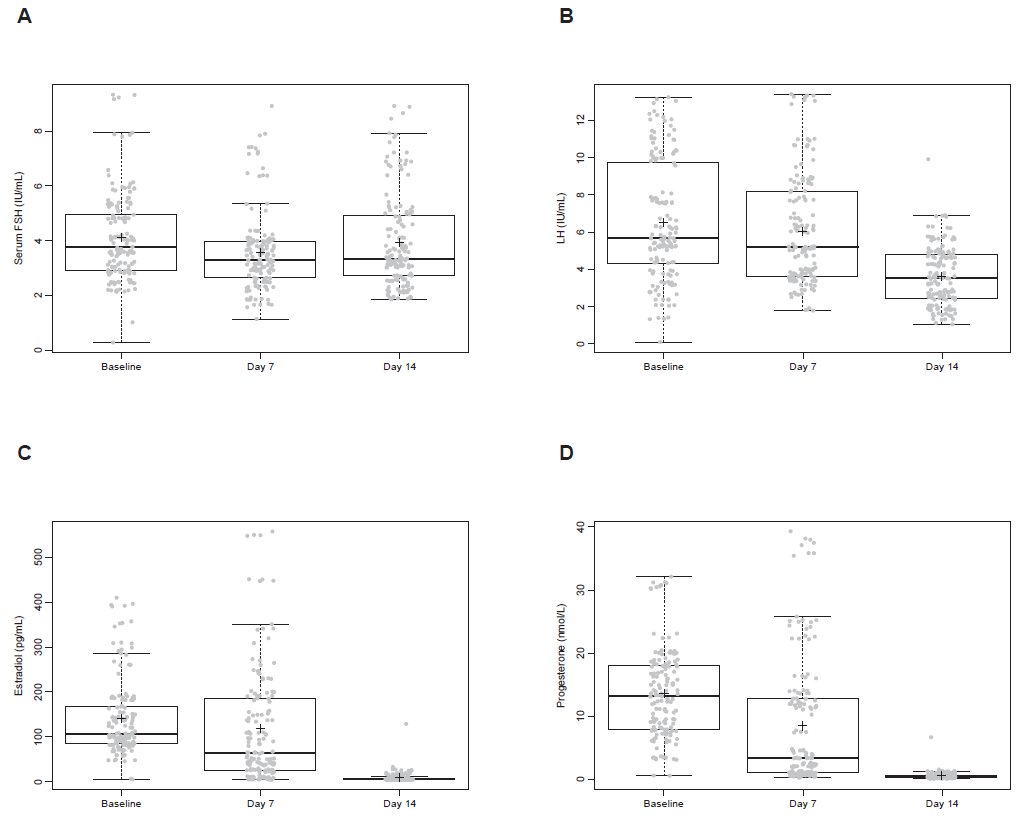


**Supplementary Table S1**

Mean ± SD and minimum–maximum serum levels of AMH, FSH, LH, estradiol, and progesterone at baseline (prior to GnRH-agonist treatment) and on days 7 and 14 during GnRH-agonist treatment.

| **Hormone^a^** | **Baseline** | **Day 7** | **Day 14** |
| --- | --- | --- | --- |
| AMH, ng/mL | 1.65 ± 0.811 0.340–3.73 | 1.39 ± 0.700  0.363–3.74 | 1.94 ± 0.865  0.585–4.45 |
| AMH, pmol/L | 11.8 ± 5.79 2.43–26.6 | 9.92 ± 5.00  2.59–26.7 | 13.9 ± 6.18  4.18–31.7 |
| FSH, IU/L | 4.22 ± 1.77 0.319–9.25 | 3.72 ± 1.58 1.17–8.91 | 4.05 ± 1.67 1.92–8.72 |
| LH, IU/L | 6.72 ± 3.66 0.100–14.8 | 6.17 ± 3.00 1.85–13.5 | 3.70 ± 1.92 1.06–9.92 |
| Estradiol, pg/mL | 142 ± 87.9 5.00–410 | 125 ± 127 5.00–551 | 10.6 ± 18.0 5.00–129 |
| Progesterone, nmol/L | 12.9 ± 7.35 0.569–32.1 | 8.90 ± 10.1 0.224–38.2 | 0.613 ± 0.914 0.089–6.67 |

^a^ Values are mean ± SD and minimum–maximum values and *n* = 52 for all measurements.

AMH, anti-Müllerian hormone; FSH, follicle stimulating hormone; GnRH, gonadotropin-releasing hormone; LH, luteinizing hormone; SD, standard deviation.

**Supplementary Table S2**

Change in AMH level at days 7 and 14 relative to baseline estimated by a linear mixed-effects model.

| **Fixed effects** | **Estimate** | ***P* value** | **95% CI** |
| --- | --- | --- | --- |
| Intercept | 1.9 | 0.1143 | –0.56, 4.36 |
| Visit day 7 | –0.28 | 0.0002 | –0.41, –0.14 |
| Visit day 14 | 0.3 | 0.0004 | 0.14, 0.45 |
| Age | –0.02 | 0.58 | –0.07, 0.04 |
| BMI | –0.02 | 0.39 | –0.07, 0.03 |
| Baseline AFC | 0.08 | 0.0015 | 0.03, 0.13 |
| **Random effects** | **Variance** | **SD** |  |
| Intercept | 0.48 | 0.70 |  |
| Visit day 7 | 0.23 | 0.48 |  |
| Visit day 14 | 0.28 | 0.53 |  |
| Residual | 0.0008 | 0.03 |  |

AFC, antral follicle count; AMH, anti-Müllerian hormone; BMI, body mass index; CI, confidence interval; SD, standard deviation.
